# Brief moderate-intensity aerobic exercise improves the executive function of Chinese undergraduates regardless of mobile phone addiction: Evidence from the antisaccade task

**DOI:** 10.3389/fpsyg.2023.849442

**Published:** 2023-03-09

**Authors:** Junyi Zhou, Zhanshuang Bai

**Affiliations:** ^1^School of Physical Education and Sport Sciences, Fujian Normal University, Fuzhou, China; ^2^Provincial University Key Laboratory of Sport and Health Science, Fujian Normal University, Fuzhou, China; ^3^Key Laboratory of Kinesiological Evaluation General Administration of Sport of China, Fujian Normal University, Fuzhou, China

**Keywords:** aerobic exercise, executive function, mobile phone addiction, antisaccade task, undergraduates

## Abstract

**Introduction:**

Previous studies have shown that brief moderate-intensity aerobic exercise can improve the executive function of healthy adults. The present study sought to examine and compare the effects of brief moderate-intensity aerobic exercise on the executive functions of undergraduates with and without mobile phone addiction.

**Method:**

Thirty-two healthy undergraduates with mobile phone addiction were recruited and randomly assigned to either an exercise or control group. Likewise, 32 healthy undergraduates without mobile phone addiction were recruited and randomly assigned to either an exercise or control group. Participants were asked to perform moderate-intensity aerobic exercise for 15 minutes for the exercise groups. The executive functions of all participants were assessed via the antisaccade task twice (i.e., pre-test and post-test).

**Results:**

The results showed that the saccade latency, variability of saccade latency, and error rate decreased significantly from pre-test to post-test for all participants. More importantly, after the 15-min moderate-intensity aerobic exercise intervention, participants in the exercise groups showed significantly shorter saccade latency than their counterparts in the control groups, regardless of whether they are with mobile phone addiction.

**Discussion:**

This result is consistent with previous studies demonstrating that brief moderate-intensity aerobic exercise can improve one’s executive function. Furthermore, the absence of significant interaction among Time, Group, and Intervention implies that the effects of brief moderate-intensity aerobic exercise on executive function are comparable between participants with and without mobile phone addiction. The present study supports the previous conclusion that brief moderate-intensity aerobic exercise can improve one’s executive function effectively, and extends it to the population with mobile phone addiction. In summary, the present study has some implications for understanding of the relationship between exercise, executive function, and mobile phone addiction.

## Introduction

Mobile phones have an enormous impact on modern lifestyles. Mobile phones are no longer simply a communication tool as they once were when they were invented. With the rapid development of mobile Internet technology and cell phones, people can use cell phones for shopping, entertainment, work, and study. China has the largest population of mobile phone users in the world. According to the latest Statistics of The 48th Statistical Reports on Internet Development in China, as of June 2021, the size of mobile phone users in China has reached 1.007 billion (China Internet Network Information Center, 2021). Young undergraduates make up a large portion of these cell phone users (Liu et al., 2017). Nowadays, mobile phones are a part of undergraduates’ lives. Moreover, since the outbreak of COVID-19, many cities in China have been under lockdown. The lockdown of the city has led to a drastic reduction in people’s outdoor activities, as the restaurants, theaters, cinemas, stadiums, gyms and other public places of entertainment were closed. This has made mobile phones a more important tool for undergraduates to entertain, socialize, and express their personal opinions on social media, and their time spent on mobile phones has increased dramatically. However, long-time mobile phone use makes undergraduates suffer a large number of negative outcomes while they bring them convenience and pleasure. Mobile phone addiction is one of the most prevalent negative outcomes (Yang et al., 2019). Mobile phone addiction have some characteristic symptoms: (1) excessive use; (2) problems with parents associated with excessive use; (3) interference with other school or personal activities; (4) a gradual increase in mobile phone use to obtain the same level of satisfaction, as well as the need to substitute operative devices with the new models that appear on the market; and (5) the need to use the mobile phone frequently, as well as emotional alterations when phone use is impeded (Chóliz, 2010).

It has been shown that about 21.3% of Chinese undergraduates suffer from mobile phone addiction (Long et al., 2016). Mobile phone addiction impairs undergraduates’ academic performance and social relationships (Liu et al., 2017; Hou et al., 2021). Moreover, mobile phone addiction could lead to many cognitive impairments, such as poor self-regulation, poor attentional control, and poor executive functions (Višnjić et al., 2018; Zhang et al., 2020).

Executive function refers to an effortful mental process needed when one pays attention to external or internal stimuli, focusing on task-relevant information and suppressing task-irrelevant information, and controlling one’s thoughts or behavior (Nigg, 2000; Diamond, 2013). Executive function deficit is one of the most critical factors contributing to mobile phone addiction (Gao et al., 2020). Studies have shown that people with higher levels of mobile phone addiction tend to have a poorer executive function, while the elevated executive function is usually associated with low levels of mobile phone addiction (Hong et al., 2012; Moisala et al., 2016; Hong et al., 2020). Therefore, exploring an effective way to enhance the executive function of college students with cell phone addiction is important.

Some researchers have found that moderate-intensity aerobic exercise can improve one’s executive function (Colcombe et al., 2004; Kamijo et al., 2009; Samani and Heath, 2018; Heath and Shukla, 2020). Moderate-intensity aerobic exercise is usually defined as exercise with an intensity of 50%–70% of one’s maximum heart rate. Previous studies have demonstrated that moderate-intensity aerobic exercise can improve executive function (Samani and Heath, 2018; Petrella et al., 2019; Heath and Shukla, 2020). Samani and Heath (2018) investigated the effect of a single-bout of brief (10 min) moderate-intensity aerobic exercise on the executive function of healthy young adults using the antisaccade task. They found that participants’ antisaccade reaction times decreased significantly after exercise intervention. Furthermore, Petrella et al. (2019) examined whether exercise intensity affects the magnitude of the benefits of exercise on the executive function of older adults. Participants completed 10-min constant load exercises at various intensities. The antisaccade task was used to assess one’s pre-and post-exercise executive function. They found that 10-min exercise benefits older adults’ executive function, regardless of whether the exercise intensity is moderate or heavy. Recently, Heath and Shukla (2020) investigated the immediate effect of a single bout of aerobic exercise on cognitive flexibility, which is one core executive function. They found that 20-min aerobic exercise can effectively reduce one’s switch cost. They suggested that a single bout of aerobic exercise can improve one’s cognitive flexibility.

These studies above indicated that brief moderate-intensity aerobic exercise could effectively improve the executive function of healthy populations. However, a study found a null effect of brief exercise on executive function. Wang et al. (2015) examined the effect of moderate-intensity aerobic exercise on executive function assessed by the Wisconsin Card Sorting Test. Young adult participants were assigned to an exercise or reading control group. They had participants in the exercise group perform a 20-min moderate-intensity aerobic exercise, and participants in the control group read books for the same time. Neither the main effect of treatment (i.e., exercise vs. reading) nor the interaction between treatment and time (i.e., pre-test vs. post-test) was observed. However, all participants performed better on WCST in the post-test than in the pre-test.

Furthermore, it is essential to note that previous studies regarding how brief moderate-intensity aerobic exercise can improve one’s executive function have potential limitations. For example, some previous studies used cognitive tasks such as the Flanker task, Go/no-go task, and WCST to assess one’s executive function. However, some researchers argued that these tasks used to assess executive function require not only executive function but also some non-executive functions such as language, visual perception (Stroop task), and even perceptual-motor skill (Flanker task). Thus, these tasks, to some extent, may not have sufficient temporal resolution to detect subtle changes in executive function.

In summary, further research is needed to investigate the effects of brief moderate-intensity aerobic exercise on the executive function of undergraduates with mobile phone addiction. The hand-and language-free nature and high temporal resolution of eye-tracking technology make it an ideal tool for assessing executive function (Petrella et al., 2019). In the present study, we used the antisaccade task to measure the inhibitory control component of executive functions. In the antisaccade task, a participant is told to inhibit an automatic, visually-guided saccade to a peripheral target, and to make an antisaccade in the opposite direction to the mirror image location of the target. If the participant fails to inhibit the reflexive saccade and makes a saccade toward the target, this is referred to as an antisaccade error. Therefore, the antisaccade task probes one’s ability to exert inhibitory control by overcoming the dominant reflexive saccade response (Kaufman et al., 2010; Meier et al., 2018). We have undergraduates with and without mobile phone addiction in the exercise groups perform a 15-min single-bout of moderate-intensity aerobic exercise *via* a cycle ergometer and assess their pre-and post-exercise executive control using the antisaccade task. Based on previous studies, we assume that brief moderate-intensity exercise can improve the executive function of undergraduates with mobile phone addiction. Specifically, we hypothesized that if brief moderate-intensity aerobic exercise does improve the executive function of undergraduates with mobile phone addiction, we should observe that participants would exhibit shorter antisaccade latencies, decreased variability of saccade latency, and lower error rates after exercise intervention. In contrast, participants in the control group would not show a significant difference in these eye movement measures between pre-test and post-test. Moreover, the effect of brief moderate-intensity aerobic exercise on undergraduates with mobile phone addiction should be larger than that on undergraduates without mobile phone addiction, and we should observe an interaction among intervention, group, and pre/post-test.

## Method

### Ethics statement

Approval to conduct this research was granted by the Ethical Committee of Fujian Normal University. All participants voluntarily provided informed consent to participate. The present study was performed in full compliance with the Declaration of Helsinki.

### Participants

Mobile Phone Addiction Tendency Scale for College Students (MPATS) was used to screen participants with mobile phone addiction. A participant with a total score of 48 or above would be classified as mobile phone addicted (Xiong et al., 2012). We screened 32 college students who scored over 48 on the MPATS as participants with mobile phone addiction. Their ages ranged from 17 to 26 years, with an average of 19.9 ± 1.77 years. These participants were randomly assigned to either the exercise or control groups (*n* = 16 in each group). To examine the difference in exercise effects between participants with and without mobile phone addiction, we screened another 32 undergraduates who scored under 48 on the MPATS as participants without mobile phone addiction. Their ages ranged from 17 to 24 years, with an average of 20.0 ± 1.84 years. Likewise, they were randomly assigned into either the exercise or control groups (*n* = 16 in each group). Each participant was paid 50 Chinese Yuan (RMB) for their participation. All participants had normal or corrected-to-normal vision, with no reported color blindness. In addition, none of them had exercise-related discomfort, cardiovascular disease, traumatic brain injury, neurological or psychiatric disorders, or been taking medication.

### Apparatus and tools

The antisaccade task was programmed in Experimental Builder (SR Research Ltd.). The materials were presented on a 17-inch DELL PC laptop (DELL VOSTRO 15; 149 resolution: 1,920 × 1,080 pixels; refresh rate: 150 Hz). Stimuli were displayed in black (RGB: 0, 0, 0) on a gray background (RGB: 153, 153, 153). Participants were seated at a viewing distance of approximately 58 cm from the computer monitor. A chin rest was used to stabilize the participants’ heads. Participants viewed stimuli binocularly while only their right eyes were monitored. Their eye movements were recorded using an Eyelink Portable Duo eye-tracking system with a sampling rate of 1,000 Hz.

#### Mobile phone addiction tendency scale for college students

Xiong et al. (2012) developed mobile Phone Addiction Tendency Scale for College Students (MPATS). The factor structure of MPATS was analyzed using exploratory factor analysis (EFA) and confirmatory factor analysis (CFA). In EFA, principal components analysis with varimax rotation was used, items whose factor loadings less than 0.4 and items with cross-loading were deleted. Factors with eigenvalues above 1 were retained, which yields four factors that explained 54.3% of the total variance. Then, Xiong and colleagues conducted EFA. Maximum likelihood estimation were used in CFA, and the four-factor model fit was estimated with following fit indices: *χ^2^* = 285.67, *df* = 98, *χ^2^/df* = 2.92, RMSEA = 0.07, CFI = 0.96, NFI = 0.94, IFI = 0.96, RFI = 0.93. The correlation coefficients between any two factors ranged from 0.24–0.61 (*ps* < 0.01). The correlation coefficients between any factor and the total score of the scale ranged from 0.55 to 0.89 (*ps* < 0.01). The Cronbach’s α coefficient of the whole scale was 0.83, and the test–retest reliability coefficient was 0.91. The Cronbach’s α coefficients for four factor were 0.85, 0.75, 0.76, and 0.79. The final version of MPATS consists of 16 items, each rated on a 5-point Likert scale, a total of 80 points. This scale contains four factors: (1) withdrawing symptoms, (2) addictive behaviors, (3) comfort brought by using mobile phones, and (4) emotional changes brought by the inaccessibility of mobile phones (Xiong et al., 2012). In the present study, the Cronbach’s α coefficient of the whole scale is 0.906, the Cronbach α coefficients for four factor were 0.717, 0.798, 0.816, and 0.617. All participants in the present study were presented with Chinese version of the MPATS. The English version of the MPATS is presented in Appendix.

### Procedure

The experimental design of the present study is a three-factor mixed design with Group (addicted vs. non-addicted) and Intervention (exercise vs. control) as between-subject factors and Time (pre-test vs. post-test) as a within-subject factor. For the exercise group, participants were asked to perform the moderate-intensity aerobic exercise using a bicycle ergometer (Ergoline, Germany) for 15 min. We monitored participants’ heart rates using a heart rate sensor (Polar, Finland). There was a 2-min warm-up period before the moderate-intensity exercise, during which participants’ heart rates did not exceed 50% of the maximum heart rate. After the warm-up, participants performed moderate-intensity (i.e., 60%–70% HRmax) exercise for 15 min. During moderate-intensity exercise, participants were asked to limit the revolution speed between 55–65 r/m. The resistance of the bicycle ergometer would be adjusted to ensure participants reach and maintain 60%–70% of their maximum heart rate. At the end of 15 min of moderate-intensity exercise, participants were given a 2-min cool-down period. For the control group, participants were asked to sit quietly and read articles from magazines or newspapers for the same time. Each participant’s executive function was examined pre-and post-intervention using the antisaccade task. The antisaccade task comprised 75 trials. Five of them were practice trials. Each trial began with a fixation cross (1° × 1°) at the center of the screen displayed for 1,000 ms. Then, the target circle (1.2°) was displayed with an eccentricity of ±10 °of visual angle in the horizontal plane for 1,500 ms (35 trials for each side), followed by an intertrial interval randomly varied between 800 and 1,200 ms. Participants were instructed to fixate on the cross to ensure that they looked at the center of the screen when the target appeared peripherally. They were also instructed to direct their gaze toward the mirror image location of the target appearing parafoveally as quickly and accurately as possible. Participants were tested individually in a quiet room with constant brightness. After reading the experimental instructions and a brief description of the apparatus, the chair’s height was adjusted to make them feel comfortable, and the eye tracker was calibrated using a 9-point calibration and validation procedure. The maximal error of validation was below 0.5 °in the visual angle. At the beginning of each trial, a black circle (0.5° × 0.5°) was presented on the center of the computer screen for drift correction. Once the participant successfully fixated on the black circle, the following stimuli were displayed. The antisaccade task lasted about 12 min.

### Statistical analysis

We used Data Viewer (SR Research Ltd.) to analyze the raw eye movement data. The following criteria for data inclusion were adopted in analyses to ensure the including eye movement data are qualified (Jazbec et al., 2006). (1) Saccade with a latency between 80 and 800 ms. (2) Saccade duration must be larger than 25 ms, and (3) saccade amplitude must be greater than 3°. This resulted in a loss of approximately 10% of the trials. Based on these criteria, the following saccade measures were derived: (1) Saccade latency, which was defined as the time elapsed from the onset of the target to the onset of the initial saccade toward the mirror image location of the target after target onset. (2) Variability of saccade latency, which was defined as the standard deviation of saccade latency. (3) Error rate, the probability that participant wrongly executes a prosaccade instead of an antisaccade.

## Results

The eye movement measures of two groups of participants in pre-and post-test antisaccade tasks are presented in Table 1. We perform a 2(addicted, non-addicted) × 2(Intervention: exercise, control) × 2(Time: pre-test, post-test) repeated measure ANOVA for each dependent variable, respectively.

**Table 1:** Mean and standard deviation of eye movement measures in antisaccade task for all groups of participants.

Group	Intervention	Time	Saccade latency	Variability of saccade latency	Error rate
Non-addicted undergraduates	Exercise	Pre-test	235 (68.7)	44.5 (22.4)	0.11 (0.111)
		Post-test	218 (73.4)	44.6 (24.2)	0.08 (0.061)
	Control	Pre-test	231 (30.6)	40.6 (19.6)	0.11 (0.083)
		Post-test	219 (34.3)	36.2 (17.1)	0.10 (0.077)
Addicted undergraduates	Exercise	Pre-test	246 (36.3)	51.5 (16.4)	0.20 (0.164)
		Post-test	231 (32.9)	44.1 (12.8)	0.15 (0.093)
	Control	Pre-test	231 (33.7)	45.0 (17.4)	0.19 (0.194)
		Post-test	231 (38.1)	40.3 (21.7)	0.09 (0.114)

For saccade latency, the ANOVA revealed significant main effect of Time, *F*(1, 60) = 22.17, *p* < 0.001, *η^2^_p_* = 0.270, but not Group, *F*(1, 60) = 0.62, *p* = 0.435, *η^2^_p_* = 0.010, and Intervention, *F*(1, 60) = 0.17, *p* = 0.682, *η^2^_p_* = 0.003. Participants showed shorter saccade latency in post-test (225 ms) than that in pre-test (236 ms). A significant interaction between Time and Intervention was observed, *F*(1, 60) = 5.08, *p* = 0.028, *η^2^_p_* = 0.078. Further analysis revealed that the saccade latency in post-test (225 ms) was significantly shorter than that in pre-test (241 ms) for exercise groups, *t*(31) = 4.73, *p* < 0.001, *Cohen’s d* = 0.84. Yet, the saccade latency in post-test (225 ms) did not differ from that in pre-test (231 ms) for control groups, *t*(31) = 1.77, *p* = 0.087, *Cohen’s d* = 0.31. The interaction among Time, Group, and Intervention was not significant, *F*(1, 60) = 1.48, *p* = 0.229, *η^2^_p_* = 0.024.

For variability of saccade latency, the main effects of Time was significant, *F*(1, 60) = 6.97, *p* = 0.011, *η^2^_p_* = 0.104. Participants showed less variability in post-test (41.3 ms) than that in pre-test (45.4 ms). The main effect of Group, *F*(1, 60) = 0.68, *p* = 0.414, *η^2^_p_* = 0.011, and Intervention, *F*(1, 60) = 1.54, *p* = 0.219, *η^2^_p_* = 0.025, were not significant. There were no significant interactions (*Fs* < 1.52).

For error rate, the ANOVA revealed significant main effect of Time, *F*(1, 60) = 13.63, *p* < 0.001, *η^2^_p_* = 0.185, and Group, *F*(1, 60) = 4.32, *p* = 0.042, *η^2^_p_* = 0.067, but not Intervention, *F*(1, 60) = 0.27, *p* = 0.604, *η^2^_p_* = 0.005. Participants showed lower error rate in post-test (0.10) than that in pre-test (0.15). Non-addicted participants (0.10) showed lower error rate than addicted participants (0.16). The interaction between Time and Group was marginally significant, *F*(1, 60) = 3.87, *p* = 0.054, *η^2^_p_* = 0.061. Further analysis revealed that the error rate in post-test (0.12) was significantly lower than that in pre-test (0.19) for addicted participants, *t*(31) = 3.87, *p* < 0.001, *Cohen’s d* = 0.68. The error rate in post-test (0.15) did not differ from that in pre-test (0.16) for non-addicted participants, *t*(31) = 1.27, *p* = 0.213, *Cohen’s d* = 0.23. The interaction between Group and Intervention was not significant, *F*(1, 60) = 0.67, *p* = 0.418, *η^2^_p_* = 0.011. The interaction among Time, Group, and Intervention was not significant, *F*(1, 60) = 1.39, *p* = 0.242, *η^2^_p_* = 0.023.

## Discussion

The present study used eye-tracking technology to examine the effect of brief moderate-intensity aerobic exercise on the executive function of undergraduates with and without mobile phone addiction. We found that participants in the exercise groups showed shorter saccade latency than control groups, regardless of whether participants are with or without mobile phone addiction. This finding supports our hypothesis that brief moderate-intensity aerobic exercise can improve the executive function of participants with mobile phone addiction. However, we did not find significant interaction among Time, Group, and Intervention. Therefore, the current results did not support our hypothesis that the effects of brief moderate-intensity aerobic exercise on executive function would be different between participants with and without mobile phone addiction.

The saccade latency and variability of saccade latency decreased significantly from pre-test to post-test which reflects practice effects. However, the significant interaction between Time and Intervention indicates that after the 15-min moderate-intensity aerobic exercise intervention, participants in the exercise groups showed significantly shorter saccade latency than their counterparts in the control groups, regardless of whether they were with or without mobile phone addiction. This result is consistent with previous studies demonstrating that brief moderate-intensity aerobic exercise can improve one’s executive function (Sibley et al., 2006; Chi et al., 2014; Samani and Heath, 2018). In addition, some previous studies revealed that saccade latency varies as a function of cognitive function (Noiret et al., 2018; Coors et al., 2021; Thomas et al., 2021). The individual with higher cognitive function tends to exhibit a shorter saccade latency. Researchers suggested that the cognitive processes underlying the antisaccade task are linked to the activity of prefrontal executive networks and frontoparietal networks (Ford et al., 2005; Everling and Johnston, 2013). Exercise can boost cortical blood flow (Ogoh and Ainslie, 2009) and increase the activity, connectivity, and density of frontoparietal areas (Voss et al., 2010; Ruscheweyh et al., 2011). Thus, the current result may suggest that exercise ‘can result in higher activity of the prefrontal executive networks and frontoparietal networks for participants. As a result, the exercise group participants’ executive functions were significantly improved, which is reflected in shorter saccade latency. Nevertheless, we did not find a significant interaction among Time, Group, and Intervention, which implies that the effects of brief moderate-intensity aerobic exercise on executive function are comparable between participants with and without mobile phone addiction.

For error rate, the significant main effect of Time indicates that all participants made fewer errors in the post-test than in the pre-test, which reflects practice effects in the antisaccade task. Additionally, the significant main effect of Group implies that participants with mobile phone addiction may have inferior inhibitory control than their counterparts, which making it more difficult to inhibit the bottom-up processes. More importantly, we found a significant interaction between Time and Group which shows that addicted participants made fewer errors in the post-test than in the pre-test, while non-addicted participants’ error rates in the pre-and post-test were comparable. This result shows that the effect of practice differed for the two groups of participants. Samani and Heath (2018) found a partially similar pattern of results. Their results showed that saccade latency decreased significantly from pre-to post-exercise assessment, while the error rate did not vary reliably from pre-to post-exercise assessment. They argue that the pattern of their results suggested that the decrease in latency does not come at the cost of making more errors and was, therefore, unrelated to the speed-accuracy trade-off. Practice effect may contribute to this result as the interval between the pre-test and post-test is short in our experiment. Therefore, repeating the antisaccade task twice in a short period may yield a substantial practice effect, which led to a significant reduction in error rate for all participants.

Our study also has important limitations. First, although this scale has been widely used in mainland China the developers of the scale have not established a norm. Therefore, we could only use the developers’ recommended criteria to distinguish between participants with or without mobile phone addiction. This could potentially affect the applicability of the current study findings. However, recent research suggests that the MPATS can be used either as a norm referenced test or as a criterion referenced test depending on the research intention (Li et al., 2019). Therefore, we believe that the use of the scale in the present study is appropriate. Second, the reliability of the MPATS can be further improved. Li et al. (2019) suggested that for specific dimensions of the MPATS, the reliability of the test can be improved by increasing the number of items in each dimension. In the present study, although the Cronbach’s α coefficient of the whole scale is 0.906, the Cronbach α coefficient for one factor (e.g., emotional changes brought by the inaccessibility of mobile phones) were only 0.617. This is consistent with Li et al.’s findings. Third, the present study did not distinguish between different types of mobile phone addiction. A recent research has shown that there are different types of mobile phone addiction, including mobile social networking addiction, mobile game addiction, mobile information acquisition addiction, and mobile short-form video addiction (Liu et al., 2022). Moreover, Liu and colleagues developed a 26-item MPATS to measure four types of mobile phone addiction. Future studies should take the type of mobile phone addiction into account according to research intention. Fourth, all participants in our study were undergraduates in university, which makes their level of education, lifestyle, and daily physical activity differ from other populations. This leads to a potential limit to the external validity of the present study. Future studies could validate the current findings in different populations. Fifth, the condition of potential gain from the brief aerobic exercise should be constrained. Some variables needed to be controlled in future research concerning the long-term effects of aerobic exercise on cognitive functions of populations with mobile phone addiction (e.g., BMI, physical fitness, level of physical activity, average screen time, etc.) because these confounding variables may interfere with the effect of aerobic exercise on inhibitory control.

The present study’s findings shed some light on our understanding of the relationship between brief moderate-intensity aerobic exercise and executive function. First, the current findings replicate the results of previous studies, supporting that brief moderate-intensity aerobic exercise does improve one’s executive function assessed by the antisaccade task (Samani and Heath, 2018; Petrella et al., 2019; Heath and Shukla, 2020). Second, many previous studies focus mainly on the effect of aerobic exercise on executive functions of the general population. The present study extends the findings of previous studies to a mobile phone addicted sample of university students, indicating that brief moderate-intensity exercise is also an effective way to improve the executive function of the general population and the population with mobile phone addiction. Third, the present study provides preliminary evidence demonstrating that brief moderate-intensity aerobic exercise can be used to improve the executive function of people with or without mobile phone addiction.

## Conclusion

In summary, the present study found that brief moderate-intensity aerobic exercise facilitates mobile phone addicted undergraduates’ performance on the antisaccade task, indicating that brief moderate-intensity aerobic exercise enhances the executive functions and attentional systems of undergraduates with mobile phone addiction. Generally, the present study has some implications for our understanding of the relationship between exercise, executive function, and mobile phone addiction.

## Data availability statement

The original contributions presented in the study are included in the article/Supplementary material, further inquiries can be directed to the corresponding author.

## Ethics statement

The studies involving human participants were reviewed and approved by Ethical Committee of Fujian Normal University. The patients/participants provided their written informed consent to participate in this study.

## Author contributions

JZ contributed to the conception and design of the study, cleaned the raw data and performed the statistical analysis, and wrote the first draft of the manuscript. ZB recruited and conducted the experiment. All authors contributed equally to the article and approved the submitted version.

## Funding

This research was supported by a grant from the Fujian Natural Science Foundation (2022J05044).

## Conflict of interest

The authors declare that the research was conducted in the absence of any commercial or financial relationships that could be construed as a potential conflict of interest.

## Publisher’s note

All claims expressed in this article are solely those of the authors and do not necessarily represent those of their affiliated organizations, or those of the publisher, the editors and the reviewers. Any product that may be evaluated in this article, or claim that may be made by its manufacturer, is not guaranteed or endorsed by the publisher.

## Appendix

Mobile Phone Addiction Tendency Scale for College Students.

**Table:**

Item					
1. Without a mobile phone for a while I will immediately check whether there are text messages or missed calls.	1	2	3	4	5
2. I prefer mobile chatting to direct face-to-face communication.	1	2	3	4	5
3. When waiting for someone I always call the phone frequently to ask where the she/he is, if not I will be anxious to play.	1	2	3	4	5
4. If I do not use the mobile phone for a long time, I will feel uncomfortable.	1	2	3	4	5
5. In class, I cannot pay attention because of phone calls or messages.	1	2	3	4	5
6. I would feel lonely if I do not have a mobile phone.	1	2	3	4	5
7. I feel more confident when I use mobile phones to communicate with others.	1	2	3	4	5
8. If the mobile phone does not ring for a period of time, I will feel uncomfortable, and subconsciously look at the mobile phone to see if there are missing calls or unread text messages.	1	2	3	4	5
9. I frequently have a hallucination that my phone is ringing or vibrating.	1	2	3	4	5
10. More phone calls and more text messages will make me feel more fulfilled in life.	1	2	3	4	5
11. I am usually worried that my mobile phone will turn off automatically.	1	2	3	4	5
12. The phone is a part of me, once I do not take it with me, I feel like I lost something.	1	2	3	4	5
13. My classmates and friends always say that I rely on my mobile phone too much.	1	2	3	4	5
14. I get anxious and angry when my mobile phone cannot connect to the network or cannot receive signals.	1	2	3	4	5
15. In class, I will often voluntarily focus on my mobile phone, which affects me in listening to the lecture.	1	2	3	4	5
16. I feel more comfortable using cell phones to communicate with others.	1	2	3	4	5

Note: 1. Very inconsistent with my case; 2. Not quite in line with my case; 3. I am not sure; 4. Basically in line with my case; 5. Very consistent with my case.
