# Supplementary material for: Brief moderate-intensity aerobic exercise improves the executive function of Chinese undergraduates regardless of mobile phone addiction: Evidence from the antisaccade task
Source: Front Psychol. 2023 Mar 9;14:849442. doi: 10.3389/fpsyg.2023.849442 (PMC10034195; doi:10.3389/fpsyg.2023.849442)
Supplement: Supplementary file 1 [file Table_1.DOCX]

There are five ‘csv’ files, including ‘addicted_latency.csv’, ‘normal_latency.csv’, ‘addicted_error.csv’, ‘normal_error.csv’, and ‘Subject information.CSV’.

Generally, in four eye movement data files:

‘sub’ column refers to the participants’ code or name.

‘group’ column refers to whether the participant is with mobile phone addiction or without mobile phone addiction.

‘prepost’ column refers to whether the data is from the pre-test or post-test.

‘exercise’ column refers to whether the participant received the exercise intervention.

The ‘addicted_latency.csv’ and ‘normal_latency.csv’ are the eye movement data files we used to calculate the saccade latency and variability of saccade latency of participants with/without mobile phone addiction. We clean the eye movement data according to the criteria we mention in the manuscript（Here's the R code for our data cleaning）.

AntiSacFix_filter <- AntiSacFix %>% filter(type == "experiment",

CURRENT_FIX_NEAREST_INTEREST_AREA_LABEL == "CrossArea",

CURRENT_FIX_INTEREST_AREA_RUN_ID == "1",

AntiSaccade == "antisaccade",

CURRENT_FIX_DURATION >= 80 & CURRENT_FIX_DURATION <= 800,

NEXT_SAC_AMPLITUDE >= 3,

NEXT_SAC_DURATION >= 25)

After the data cleaning, we calculated the fixation duration (CURRENT_FIX_DURATION column in the files) in the Cross Area at the appearance of the parafoveal target in the pretest or posttest for each participants in different groups (normal/addicted). We also calculated the variability of saccade latency (the standard deviation of CURRENT_FIX_DURATION) for each participants.

The ‘addicted_error.csv’ and ‘normal_error.csv’ are the eye movement data files we used to calculate the error rates of participants with/without mobile phone addiction. We clean the eye movement data according to the criteria we mention in the manuscript（Here's the R code for our data cleaning）.

AntiSacError_filter <- AntiSacFix %>%

filter(type == "experiment",

CURRENT_FIX_NEAREST_INTEREST_AREA_LABEL == "CrossArea",

CURRENT_FIX_INTEREST_AREA_RUN_ID == "1",

CURRENT_FIX_DURATION >= 80 & CURRENT_FIX_DURATION <= 800,

NEXT_SAC_AMPLITUDE >= 3,

NEXT_SAC_DURATION >= 25)

After the data cleaning, we calculated the ratio of incorrect trials to the total number of trials for each participants in the antisaccade task to derive the error rate.
